# Ethical implications for children’s exclusion in the initial COVID-19 vaccination in Ghana

**DOI:** 10.1080/11287462.2023.2168170

**Published:** 2023-01-23

**Authors:** Samuel Asiedu Owusu

**Affiliations:** Directorate of Research, Innovation and Consultancy, University of Cape Coast, Cape Coast, Ghana

**Keywords:** Children, COVID-19, risk, vaccination, ethics, Ghana

## Abstract

Bioethics provides various models of fair allocation of scarce health resources like COVID-19 vaccines. Even though these models are grounded in some ethical principles like justice and beneficence, there were severe inequalities in global access to COVID-19 vaccines. In Ghana, about 21.5 million COVID-19-doses have been administered but comprise mainly members of the adult population. As a result, ethical issues related to vaccinating children have been largely ignored in the country. This paper explores some of the ethical implications related to children’s exclusion in the initial COVID-19 vaccination programs in Ghana. It provides a general overview of the COVID-19 pandemic in Ghana and how it related to children and discusses the risks to which Ghanaian children were exposed by delaying their COVID-19 vaccination. A guide to facilitating the full rollout of COVID-19 vaccination in Ghana for children has been proposed that indicates that a fair vaccine distribution for children should prioritize children on admission at health facilities, those diagnosed with severe underlying health conditions, and children who could play an instrumental role in promoting vaccine uptake. It concludes that children must not be placed at the peripheries of the COVID-19 vaccination program in Ghana.

## Introduction

Some COVID-19 mitigation measures were implemented in various countries including Ghana to contain and mitigate the public health challenges that were posed by the pandemic but most of the measures initially focused on the adult population. This paper discusses some ethical implications for excluding children from the initial COVID-19 vaccination in Ghana and provides a guideline for incorporating children in future vaccination programs in the country. Children are defined as comprising all members of human descent in Ghana who are currently sentient and below 18 years of age (UNICEF, 1989; Republic of Ghana, 1992). The paper starts with an overview of the COVID-19 pandemic and how it affected children. This is followed by an argument that the delay in involving children in the initial COVID-19 vaccine rollout in Ghana posed significant risks for the children and public health. It ends with a proposed guideline for a nationwide COVID-19 vaccine distribution for Ghanaian children. Objections to some of the claims have been considered as well as their corresponding replies provided.

## An overview of the COVID-19 pandemic and children

Sentient human beings, at least those who minimally care about the nuances and implications of public health pandemics, were awakened in November 2019 by the news of the emergence of a novel SARS-CoV-2 coronavirus disease (COVID-19). This disease was later declared by the World Health Organization Emergency Committee as a global health emergency based on the growing case notification rates in China and other international locations (Velavan & Meyer, 2020). The rapid infection and mortality rate as well as the consequential ravages of COVID-19 have been vividly felt and reported worldwide (Asante & Mills, 2020; Bostan et al., 2020; Ekumah et al., 2020; Rahman et al., 2021; Upoalkpajor & Upoalkpajor, 2020). At its onset, the potential public health challenges that were to be associated might have appeared nontragic, however, the pandemic disrupted almost every aspect of human endeavor due to its associated far-reaching public health measures like social distancing, lockdowns, closure of schools and restrictions of most outdoor activities to contain its spread (Asante & Mills, 2020; Rahman et al., 2021; Upoalkpajor & Upoalkpajor, 2020). The COVID-19 infection and mortality rates have been disproportionately felt by some categories of human population and have delved disproportionate toll on adults than children. Even among the adult population, COVID-19 gravely affected the aged and those with some underlying health conditions (Carethers, 2021) and racial groups (Mackey et al., 2021). In the United States of America, for instance, Mackey et al. have reported that African American/Black and Hispanic populations experienced disproportionate higher rates of infection and related mortality than people with other racial backgrounds. It is also noteworthy to point out that COVID-19 affected more essential and frontline workers (Lancet, 2020) than the other essential workers.

The high COVID-19 infection and mortality rates led to extensive moral debate propositions of various ethical frameworks to guide fair distribution of the limited health resources including COVID-19 vaccines. For instance, Emanuel et al. (2020) proposed that for settings of absolute scarcity, allocation of resources in pandemics ought to include four fundamental values consisting of maximizing benefits produced by scarce resources, treating people equally, promoting and rewarding instrumental value and giving priority to the worst off. These values informed their framework that advocated that priority should aim at both saving the most lives and at maximizing improvements in individuals’ post-treatment length of life. Similarly, critical COVID-19 interventions in the form of testing, distribution of personal protective equipment, intensive care unit beds, ventilators and vaccines should prioritize frontline healthcare workers and other essential workers who keep critical infrastructure operating. Other proponents like Jecker et al. (2021) have asserted that a just distribution of COVID-19 vaccines must prioritize frontline and essential workers, people at high risk of severe disease or death, and people at high risk of infection. Despite these ideal and well-thought through moral propositions, the distribution patterns of COVID-19 vaccines could be described as unjust. For instance, while some countries were yet to achieve high vaccine coverage rates in the highest and high priority-use groups (World Health Organization, 2022), others have faced vaccine hesitancy (Altindis, 2022; Bansal, 2022; Sharma et al., 2021; Wells & Galvani, 2022) despite the availability of vaccines.

Children experienced relative lower COVID-19 morbidity and mortality rates in most parts of the world and they appeared to be the population category that was least planned for in terms of COVID-19 vaccinations. Incidentally, some children experienced fears, physical and social isolation, abuse and neglect as well as disruption in their sleep patterns (Imran et al., 2020) due to the interruptions to most of their routine activities like school closures and outdoor playing grounds (Roje et al., 2020) as a result of the COVID-19 pandemic. In addition, some children have experienced lowered standard of living due to their primary caregivers’ reduced standard of living as a result of the severe economic challenges occasioned by the pandemic. Despite these monumental harms experienced by children, most policies, interventions and discourses about COVID-19 mitigation measures including vaccination programs, at least in Ghana, disproportionately tilted in favor of adults (Sam-Agudu et al., 2022). Clinical trials of children-centered COVID-19 vaccination in Ghana were rolled out in November 2021 for minors aged 15 and 17 (Annoh, 2021) while the vaccination for the adult population commenced in March of the same year. Even among the adult Ghanaian population, clinical trials of vaccinations for novel pandemics like Ebola have not received wide appeal. Already, the proposed clinical trial of a vaccine for Ebola was abandoned due to some factors including the perception that the vaccine might give trial participants Ebola as a side-effect (Kummervold et al., 2017).

It is widely permissible to include children in vaccines developed through the traditional system but exposing them to the relatively new COVID-19 vaccines should, and justifiably so, raise strong moral objections. Should we stick to the widely traditional system of certifying the safety and efficacy of newly developed vaccines for children after strong evidence from full-scale trials or, in the wake of uncertainties about pandemics like COVID-19, we should adopt a middle-ground approach? The next section presents arguments to justify that the exclusion of Ghanaian children from the initial COVID-19 vaccination was morally unjust and that their inclusion would have provided instrumental value for attaining herd immunity in the fight against the pandemic and a roadmap for tackling future novel global pandemics.

## Risk for delaying vaccine research involving children

The high COVID-19 infection and mortality rates largely affected the adult population but children have also experienced some of its socio-economic impacts (World Health Organization, 2022). Although it has been reported that full vaccination with the Pfizer-BioNTech, Moderna or AstraZeneca-approved vaccines provides substantial protection against some COVID-19 variants (Nasreen et al., 2021), public health experts are yet to authoritatively recommend full discontinuation of COVID-19 enhanced health and safety precautions (Tufekci, 2021). With the current general exclusion of most children from COVID-19 vaccination in Ghana, public health experts will have to utilize additional scarce health resources to implement specific children-centered mitigation measures instead of using the current opportunity to rope in children in the vaccinations. For instance, adults may appreciate and abide by social distancing protocols but this measure may not appeal to children who are known to be more mobile and activity oriented. On the other hand, children per se may be willing to volunteer to participate in future COVID-19 vaccination but there is also empirical evidence, as reported by Goldman et al. (2021), that parental interest in taking part in COVID-19 vaccine trials may disallow them to get their children vaccinated. With the current uncertainty regarding adults acceptance of COVID-19 vaccines (Acheampong et al., 2021; Agyekum et al., 2021; Alhassan et al., 2021; Kyei-Arthur et al., 2022), it may require more resources and effort to vaccinate children in future pandemics like COVID-19 (Kyei-Arthur et al., 2022).

Related is the effect of the emergence of a new COVID-19 variant or another pandemic disease that may affect only children (Rasaily & Mathur, 2017) and for which it was expedient to have involved them at the early stages in Ghana’s vaccination programs. Their involvement would have allowed for early gathering of the needed empirical data that would have fed into the broad national pandemic mitigation architecture. Excluding the children from the initial vaccination process violated some key ethical principles such as justice, beneficence, nonmaleficence and personal autonomy (Beauchamp, 2008; Beauchamp & Childress, 2019; United States National Commission for the Protection of Human Subjects of Research, 1978). The principle of justice, for instance, requires equitable distribution of research costs and benefits through careful selection of research participants that considers both individual and collective benefits as well as burdens. Excluding children, who constitute more than a third of Ghana’s population (Ghana Statistical Service, 2022), from vaccine research might be akin to intentionally targeting them for potential harm. Furthermore, this exclusion also runs contrary to the ethical principle of beneficence which calls for the protection of persons from harm and security of their well-being (United States National Commission for the Protection of Human Subjects of Research, 1978) and the core tenants of the Ghana Children’s ACT which guarantees some basic rights to children including immunizations (The Children’s Act 1998 (Act 560), 1998).

The inclusion of children in the initial COVID-19 vaccinations would have also enhanced the value of obtaining broad acceptability of future authorized emergency vaccines for novel pandemics. A vaccine for a future novel pandemic reminiscing that of COVID-19 would certainly receive emergency authorization from state institutions such as the Ghana Food and Drugs Authority (Government of Ghana, 2012). The early involvement of Ghanaian children in the COVID-19 vaccination program would have had the potential of registering a higher acceptance rate for future vaccines, reduced expenditure on public campaigns for vaccine acceptance and received the backing of other stakeholders who would lend support to public health campaigns for children’s vaccinations. An objector may indicate that there would be no need to re-invent the wheel since adults would offer the needed roadmap to facilitate children adoption of vaccines based on the premise that most decisions for children such as career choice (Owusu et al., 2021) are made by adults. What may be missing is the fact that COVID-19 has and continues to defy some long-held public health interventions which may require new strategies to mitigate children-targeted future pandemics. Indeed, there could be a possibility that some of the existing public health measures like nose mask mandates (Nguyen, 2020) and vaccination passports (Jeanrenaud et al., 2021) may not work well for a future pandemic variant that would infect children in a scale that COVID-19 has infected and affected the adult population.

Closely related to the unfair exclusion of children from the initial COVID-19 vaccination in Ghana is also the argument that it is morally acceptable to give children a fair chance to contribute in routing the COVID-19 pandemic. Children, irrespective of their vulnerabilities or frailties, contribute significantly to our understanding of disease etiologies and treatments (World Health Organization, 2022). They have been integral in traditional vaccine research studies and have been invaluable resources in our understanding of the science of some pandemics. They have discharged and continue to discharge their beneficent ethical duties to the development and approval of vaccines but their non-involvement in the initial COVID-19 vaccination in Ghana may gravely undermine our efforts at completely routing out COVID-19 from the country and possibly pitch children as being maleficent. Although in Ghana, relatively few children have been vaccinated with the Pfizer-manufactured COVID-19 vaccine (Annoh, 2021) neither is any child overtly seen as playing frontal roles in public health communication or advocacy programs in support of COVID-19 vaccination roll out, potential risk and benefits, fight against hesitancy and preparedness for future pandemics. Indeed, all the current public health communication figures are adults but, at least, some public outdoor performances by children for children and with children could have commenced in Ghana as peer education avenues to get children involved in the vaccination exercise.

A plausible explanation might be that children may not be fully capacitated to act as moral agents in shaping adult decisions on some important ethical issues such as participation in vaccinations. Indeed, most of the adult COVID-19-positive symptoms were asymptomatic in nature. There might also be several children who might have also tested positive for the virus but not reported because of their continued exclusion from the initial COVID-19 testing and research in Ghana except the very few who were tested for some purposes like requirements for international travels. There is also a significant traction in an argument that may view children’s involvement in the COVID-19 vaccination as constituting a wasteful utilization of limited health resources. Indeed, Emanuel and his colleagues argued that priority in allocation of scarce resources should aim at saving the most lives (Emanuel et al., 2020). It may appear absurd to dissipate the scarce resource on children who are least reported to be infected with COVID-19 (World Health Organization, 2022) but it is also unethical to overly invest in adult research or disproportionally skew limited health investments towards adults as it existed in Ghana with the initial rollout of the COVID-19 vaccinations.

The ethical principle of respect for persons requires for the protection of research participants with diminished autonomy (United States National Commission for the Protection of Human Subjects of Research, 1978). Such diminished autonomous persons are deemed incapable of self-determination and would require extensive protection including exclusion from certain high-risk research activities or provision of adequate information to enable them to make informed decision to participate or refuse participation in some activities (The Children’s Act 1998 (Act 560), 1998; UNICEF, 1989). These provisions, perhaps, might have led to the exclusion of children from the initial COVID-19 vaccinations in Ghana. What is missing from this line of thought, however, is the fact that there are also children, at least in Ghana, who are very capable of providing valid legal evidence, act as credible witnesses or have valid moral status in certain types of deliberations like those that concern their medical care (Baker, 2013; Parliament of the Republic of Ghana., 1960, 1975).

The World Health Organization issued an interim statement on COVID-19 vaccination for children in August 2022 (World Health Organization, 2022) which urged member countries to consider individual and population benefits of immunizing children in their formulation and implementation of COVID-19 immunization policies and programs. The statement recommended a vaccination system that will account for prioritization to fully protect the highest risk subgroups of the population and the need for children to continue to receive the recommended childhood vaccines for other infectious diseases. Perhaps the uncertainties that surrounded the COVID-19 pandemic informed the inaction of Ghanaian public health practitioners to involve children in the initial vaccination program but, as rightly pointed out by the Organization in the application of its precautionary principle, this constraint can result in implementing remedial actions after a hazard has been caused as manifested in the millions of children worldwide who have suffered various nervous system damage, diminished mental capacity and limited ability to make a living (Martuzzi & Tickner, 2004). Children constitute a high proportion of the Ghanaian population (Ghana Statistical Service, 2022) and for which their involvement in the fight against the COVID-19 pandemic cannot be underrated. It is expected that Ghanaian public health officers will embrace the call by the World Health Organization and position children at the forefront of the on-going COVID-19 vaccination programs in Ghana. The last section of this paper outlines a guideline that could be adopted to facilitate COVID-19 vaccination in Ghana in addition to serving as a framework for future novel pandemics.

## Proposed guideline for COVID-19 vaccination for Ghanaian children

The global public health challenges that have been associated with the emergence of COVID-19 has clearly extended the limits of health resources to meet demands. Aside the disproportionate large patient to healthcare workers ratio as a result of the pandemic in most countries, there is also a high disproportional ratio of health equipment or supplies to patient’s ratio which legitimizes the need to ration COVID-19 health resources based on an acceptable ethical distribution guideline.

Clearly, COVID-19 infection among Ghanaian children will severely affect children with some existing underlying health conditions, which is similar to the case with most adults who died from the virus. While the specific conditions may vary for children and adults, it is proposed that identifiable vulnerable children such as those who are unwell and on admission or children presented for out-patient treatments or post-natal services at all recognizable health facilities in Ghana should be prioritized for COVID-19 vaccination. This proposed priority category is based on the premise that such children already have weak immune systems or antibodies to withstand the virus as well as being pre-exposed to the virus by infected adult caregivers, adult patients or frontline health workers.

The next priority category should consider competent children who provided instrumental value during the search for an efficacious COVID-19 vaccine. This category may comprise children who participated in vaccine research, celebrities who publicly educated their peers about the pandemic or other identified competent children who served as role models for other children during the pandemic. Besides rewarding them for their varied instrumental role in containing the virus, it will also serve as a means of identifying and nurturing children public health ambassadors in Ghana. Global children environmental activists such as Greta Thunberg and Licypriya Kangujam (Annamalai, 2021; Jung et al., 2020; Kühne, 2019; Sabherwal et al., 2021; Thunberg, 2019) have risen to this challenge and provided some insights for constructive engagements of children celebrities. Prioritizing children’s celebrities in Ghana for COVID-19 vaccination would serve as avenues for demystifying some of the doubts or misconceptions about COVID-19 vaccination process.

Having served these cohorts of relatively few children, the stage would then be set to target children in the general population who usually congregate at various public and private spaces such as schools, playgrounds, chapels and households. Children are greatly relational beings and by virtue of their socialization processes, usually found in congregated spaces. They socialize through their educational institutions, religious places and play grounds (Bronfenbrenner, 1979). These places offer ideal opportunities to reach out to most children with relative minimal health resources. Such congregated environments also facilitate the consenting processes since, for example, a school-authorized representative can consent for more children to be vaccinated unlike household settings where each child and corresponding consenting adults have to be reached individually. The final prioritized batch of the children to receive the vaccines would be left with the few individuals who might have dropped out of school or engaged in other individualized activities and could be reached at the household level.

This proposed prioritization schema is not immune to some overt ethical flaws including it being viewed as exclusionist for rating some children for prioritized services over others as well as it being inherently coercive and paternalistic in nature. The state has a responsibility to protect its vulnerable population like children. This duty of care may not exactly follow measures that have been deployed for autonomous adults. An adult may have a relative justifiable reason to hesitate decisions on adoption of new vaccines but children will certainly require the state or adult directions to facilitate such decision-making processes. The state may not be faulted for discharging its legitimate rescue of care duties to its vulnerable citizens.

This guideline also fits in the Ghanaian context for two principal reasons. There is already a national recognition of the inputs of children in national policy formulations and programming (Ministry of Gender, Children and Social Protection, 2015; Ministry of Women and & Children’s Affairs, 2004; Republic of Ghana, 1992; UNICEF, 1989). Existing traditional proverbs such as *if a child learns how to wash his hands, he eats with adults* affirm the country’s affinity for children on some critical national issues such as COVID-19 vaccinations.

## Conclusion

Society is not oblivious to its moral duties to provide quality care to citizens including provision of enhanced protection and care for the vulnerable in society. The novel COVID-19 pandemic has gravely altered society’s response to this duty of care obligation. Sadly, it forced society to skew its vaccination resources in disfavor of children. This ought not to continue since the emergence of a new variant of the virus may be catastrophic for children survival and well-being. The Ghanaian society cannot continue to place children at the peripheries in its on-going COVID-19 vaccinations. Children should and ought to be at the center stage: they could play some instrumental role to support the science of the pandemic. For such children who would rise up to the challenge, their instrumental value should be rewarded by prioritizing them, along with other identifiable children with underlying health conditions, in the roll out of future novel pandemic vaccinations. At least, this can be implemented in Ghana.

## Data Availability

Data sharing is not applicable to this article as no new data were created or analyzed in this study.
